# Declarative referential gesturing in a wild chimpanzee (*Pan troglodytes*)

**DOI:** 10.1073/pnas.2206486119

**Published:** 2022-11-14

**Authors:** Claudia Wilke, Nicole J. Lahiff, Kris H. Sabbi, David P. Watts, Simon W. Townsend, Katie E. Slocombe

**Affiliations:** ^a^Department of Psychology, University of York, York, YO10 5DD, United Kingdom;; ^b^Department of Comparative Language Science, University of Zurich, CH-8050 Zurich, Switzerland;; ^c^Centre for the Interdisciplinary Study of Language Evolution, University of Zurich, CH-8050 Zurich, Switzerland;; ^d^Department of Anthropology, Tufts University, Medford, MA 02155;; ^e^Department of Anthropology, University of New Mexico, Albuquerque, NM 87131;; ^f^Department of Anthropology, Yale University, New Haven, CT 06511;; ^g^Department of Psychology, University of Warwick, Coventry, CV4 7AL, United Kingdom

**Keywords:** chimpanzee, declarative gesture, shared attention, referential communication, showing

## Abstract

Humans are argued to be unique in their ability and motivation to share attention with others about external entities—sharing attention for sharing’s sake. Indeed, in humans, using referential gestures declaratively to direct the attention of others toward external objects and events emerges in the first year of life. In contrast, wild great apes seldom use referential gestures, and when they do, it seems to be exclusively for imperative purposes. This apparent species difference has fueled the argument that the motivation and ability to share attention with others is a human-specific trait with important downstream consequences for the evolution of our complex cognition [M. Tomasello, *Becoming Human* (2019)]. Here, we report evidence of a wild ape showing a conspecific an item of interest. We provide video evidence of an adult female chimpanzee, Fiona, showing a leaf to her mother, Sutherland, in the context of leaf grooming in Kibale Forest, Uganda. We use a dataset of 84 similar leaf-grooming events to explore alternative explanations for the behavior, including food sharing and initiating dyadic grooming or playing. Our observations suggest that in highly specific social conditions, wild chimpanzees, like humans, may use referential showing gestures to direct others’ attention to objects simply for the sake of sharing. The difference between humans and our closest living relatives in this regard may be quantitative rather than qualitative, with ramifications for our understanding of the evolution of human social cognition.

Human infants as young as 10 to 13 mo old start to produce protodeclarative gestures in the form of showing or holdouts, giving, and pointing (1). Infants use these referential gestures both imperatively, with the goal of using the recipient as a social tool to act on the object, and declaratively, with the goal of directing the recipient’s attention to a common focus (2). The frequency of holdout or showing gestures predicts the subsequent frequency of pointing gestures (3), and experiments have shown that infants typically produce holdout gestures with the declarative intention of eliciting shared attention with the adults regarding the object (4)—“sharing for sharing’s sake” (ref. 3, p. 584). In contrast, great apes are only known to produce referential gestures imperatively to request objects from human caregivers or conspecifics (5, 6) or to request grooming in specific locations (7, but see ref. 8). This apparent species difference has fueled the argument that the motivation and ability to share attention with others is a human-specific trait with important downstream consequences for the evolution of our complex cognition and success as a species (6).

## Results

**Fig. 1.**
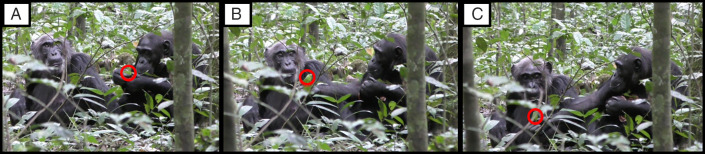
Photo series of Fiona (on the right) showing the leaf to Sutherland (on the left; the red circle shows the location of the leaf). (*A*) Fiona leaf grooming while Sutherland sits next to her, not paying visual attention to the leaf grooming. (*B*) Fiona holds the leaf toward Sutherland, and Sutherland shifts her gaze toward it. (*C*) Fiona fully extends her arm and holds the leaf directly in front of Sutherland. Sutherland noticeably moves her head and gaze to look directly at the leaf. Fiona then resumes leaf grooming.

We recorded an instance of a referential showing gesture between conspecifics in the context of leaf grooming in the Ngogo chimpanzee community, Kibale National Park, Uganda that seems to be produced declaratively. During self-grooming or social grooming, groomers occasionally pluck leaves that they manipulate with their fingers and mouths as if grooming them while also peering closely at them (9). They may be inspecting ectoparasites (e.g., ticks) they have placed on the leaves (10), but the function of leaf grooming remains unexplored in this community. The event described here involved a mother/adult daughter dyad. Adult female Fiona was sitting next to her mother Sutherland, whom she had been grooming. Fiona plucked a leaf from a small sapling and started leaf grooming. Sutherland’s attention was focused elsewhere while Fiona did this (Fig. 1 and Video S1), and after grooming the leaf for several seconds, Fiona held it out toward Sutherland. She repositioned her arm when the initial holdout did not elicit a response (Fig. 1). Once Sutherland attended to the leaf by fully orienting her eyes and head toward it, Fiona retracted it and continued leaf grooming (Video S1 and *SI Appendix*).

The movements of this behavior are in line with the definition of showing or “holdouts” in human infant literature (e.g., ref. 3). Using the operational definitions of the most recent research on infant showing and giving (11), this gesture would be coded at least as an incipient show and potentially, as a fully formed conventional show. Incipient gestures are those that are plausibly part of the developmental trajectory toward the emergence of the conventional gesture form. Moreover, Fiona showed persistence with her gesturing (indicative of intentional signaling) (12), moving the leaf closer to Sutherland and more into her line of sight until Sutherland clearly adjusted her head to follow the movement of the leaf. Although Sutherland dropped her gaze to the leaf when Fiona first extended her arm, this may not have been clear from Fiona’s perspective, and head direction could have been a more reliable indicator for her. Once Sutherland had clearly seen the leaf, Fiona ceased gesturing, suggesting that the goal of Fiona’s gesturing behavior was simply to get Sutherland to attend to the leaf (12).

However, unambiguously establishing the communicative intent of nonlinguistic signalers is challenging, particularly from a single observation. Further observations of showing behavior, ideally from multiple individuals, will be key to confirming this putative communicative goal (13). While this was the only instance of showing we observed, we examined 84 additional leaf-grooming events to explore Fiona’s likely motivation for her gesture further. These 84 video-recorded leaf-grooming events came from *n* = 37 leaf groomers from the Ngogo and Kanyawara chimpanzee communities, where at least 1 other individual was within 1 m of the leaf groomer (to mirror the Fiona–Sutherland context) (*SI Appendix* has details of the dataset, coding, and additional descriptive results). First, in support of the argument that Fiona’s goal was simply to get her mother to attend to the leaf she was grooming, simultaneous visual attention on the leaf occurred between the leaf groomer and at least one observer in 60 of 74 events (81.1%; *n* = 32 leaf groomers) where at least one group member was within 1 m and in a position to see the leaf grooming. In the Fiona–Sutherland example, Sutherland was not paying attention to Fiona’s leaf grooming, potentially creating the motivation to gesture and show the leaf in order to gain Sutherland’s attention and to facilitate simultaneous visual attention to the leaf (or ectoparasite).

Second, as chimpanzees eat both leaves and parasites, we considered whether this could be an attempt at active food sharing. However, if food sharing was Fiona’s primary motivation, one might expect her to relinquish possession of the leaf (giving or dropping the leaf for Sutherland), and this did not occur. Additionally, the species of leaf that Fiona showed Sutherland is not included in the chimpanzee diet at Ngogo (14), and in 66 leaf-grooming events where at least one subadult/adult was within 1 m, these observers never touched, took, or ate any part of the leaf. Furthermore, in the 61 events (*n* = 30 leaf groomers) where we could record whether the leaf groomers ate the leaf they were grooming, they did not do so. We, therefore, consider it highly unlikely that Fiona was trying to offer or give the leaf to Sutherland to eat.

Third, if leaf grooming is typically used imperatively to request or initiate a dyadic social activity, such as grooming or play, Fiona might have gestured to prompt one of these outcomes. However, we found no evidence that leaf grooming is used to elicit social grooming or play from a partner. In 58 events (*n* = 30 leaf groomers) where the leaf groomer’s behavior was visible both immediately (5 s) before and after leaf grooming, the leaf groomer engaged in a new social grooming or playing interaction after only 5 of these 58 events (*SI Appendix* has further details). Overall, there were no consistent differences between the leaf groomer’s behavior before and after leaf grooming, with social behaviors (social grooming, play) being more frequent before than after. This indicates that leaf grooming is not reliably used imperatively to elicit grooming or play from a partner, making it unlikely that Fiona gestured to request such an outcome.

## Discussion

Establishing communicative goals in nonlinguistic beings is challenging, and in nonhuman primate gestural research, multiple observations are typically used to identify the signaler’s putative goal (13). There is inevitably more uncertainty in identifying a signaler’s goal with a single observation, as satisfaction with the receiver’s response and a failed communicative attempt are difficult to disentangle. However, we explored other leaf-grooming events at Ngogo and in a second chimpanzee community in the same population (Kanyawara) to inform our understanding of the likely goal of the signaler in this case. Our results were consistent with the argument that Fiona gestured declaratively to show her mother, Sutherland, the leaf and that her gesture led to simultaneous attention to the leaf. We found that simultaneous attention occurred in over three-quarters of leaf-grooming events, yet it was absent in the Fiona–Sutherland interaction prior to the gesture. In contrast, it seems unlikely that Fiona gestured to engage in food sharing or to initiate dyadic play or grooming interactions. This observation represents a promising example of “sharing attention for sharing’s sake” in wild nonhuman apes.

Future research should endeavor to identify more observations of showing behavior, ideally from multiple individuals, to confirm that chimpanzees sometimes communicate with the goal of sharing attention (13). Several aspects of the Fiona–Sutherland interaction provide hints as to where such future research may find further examples of showing and other protodeclarative gestures in one of our closest living relatives. Importantly, the behavioral context here was nonurgent and noncompetitive; it did not involve feeding, mating, aggression, or a response to any threats. Additionally, Fiona was interacting with her mother, with whom she shared a close social bond. Our observation suggests that in highly specific social conditions, wild chimpanzees, like humans, may be motivated to communicate cooperatively and share interest and attention simply for the sake of sharing. If so, this raises the question of whether differences between humans and chimpanzees in the ability to engage in cooperative communication are quantitative rather than qualitative, with ramifications for our understanding of the evolution of human social cognition.

## Materials and Methods

Data were collected from the Kanyawara and Ngogo chimpanzee communities in Kibale National Park, western Uganda (*SI Appendix*, *Methods* has study site details). Video data were collected with Panasonic HDC-SD40/60/90 and Panasonic HC-V750K/VX980 camcorders over six study periods from 2013 to 2020 (*SI Appendix*, *Methods*). Behaviors of interest were coded from leaf-grooming videos by C.W. A subset of videos was coded by an independent research assistant blind to the research questions, and a high mean Cohen’s kappa score (0.91) indicated that the videos had been reliably coded (*SI Appendix*, *Methods*).

This study complied with the Association for the Study of Animal Behaviour/Animal Behavior Society guidelines for the use of animals in research. Ethical approval for data collection was granted by the Biology Animal Welfare and Ethics Review Body (University of York, York, United Kingdom). The Ugandan Wildlife Authority and the Ugandan National Council for Science and Technology granted permission to collect data in Uganda.

## Supplementary Material

Supplementary File

Supplementary File

## Data Availability

All study data are included in the article, the supporting information, or the data file deposited on the Open Science Framework (OSF): https://osf.io/ybgdx/?view_only=7e03c4262fba4497a8ee5ac163c93e61, (15).
