# Supplementary material for: Declarative referential gesturing in a wild chimpanzee (Pan troglodytes)
Source: Proc Natl Acad Sci U S A. 2022 Nov 14;119(47):e2206486119. doi: 10.1073/pnas.2206486119 (PMC9704713; doi:10.1073/pnas.2206486119)
Supplement: Supplementary File [file pnas.2206486119.sapp.pdf]

Supplementary Information Appendix

ADDITIONAL METHODS

Study site and subjects

Data were collected from the Kanyawara and Ngogo chimpanzee communities in Kibale National Park, western Uganda. Kibale is a mid-altitude tropical moist forest that includes anthropogenic grasslands, papyrus swamps, and young forest regenerating from past human disturbance along with old growth forest. Whilst the Kanyawara group's territory is bordered by agriculture and occupied villages (1), the Ngogo study area, which is approximately 10km away lies entirely within the forest. Detailed descriptions of the study areas are provided by (2) and (3).

Research on the chimpanzees of the Kanyawara community started in 1987, and has been continuous since (4). The group comprised 45-55 individuals between 2013 and 2019 (5). We collected leaf-grooming data on 14 male and 10 female chimpanzees ranging in age from 6 to ~48 years. The Ngogo community has been observed since 1978 with intensive and continuous study beginning in 1995 (6). At the time of study, the community had recently split into Ngogo Central and Ngogo West (7), with approximately 120 individuals in the new central community and 80 in the western community. Data were collected on 5 male and 8 female individuals ranging in age from 5 to ~64 years.

Data collection

Data were collected at Kanyawara in six study periods: February - May 2013 by CW, June 2014 - March 2015 by CW; January to August 2015 and 2016 by KHS; July 2016 – September 2016 by NL; January 2018 - October 2018 by CW and NL. Ngogo data were collected between January 2018 – March 2020, by CW and NL.

Video data were collected on focal individuals in a range of behavioural contexts. Leaf-grooming was not a context of interest at the time of data collection, but careful post-hoc screening of the focal videos revealed the cases we report here.

Leaf-grooming coding

Once videos containing leaf-grooming had been identified, these were coded for the following variables: 1) leaf-groomer ID, 2) start and end time of leaf-grooming, 3) IDs of individuals within 1m who would be able to observe the leaf-grooming, without having to move their torso, 4) IDs of individuals within 1m who would not be able to observe the leaf-grooming without moving their torsos, 5) IDs of individuals within 1m who peered at the leaf (face <30cm from leaf), 6) IDs of individuals within 1m who watched the leaf-grooming (face >30cm from leaf), 7) leaf-groomer behaviour immediately (5s) before leaf-grooming started, 8) leaf-groomer behaviour immediately (5s) after leaf-grooming ended, 9) whether the leaf-groomer ate the leaf.

Inter-coder reliability

Inter-coder reliability was assessed for all the coded behaviours outlined above. CW coded all 84 leaf-grooming events, and an independent research assistant blind to the research questions coded 13 of the same events (15.5%). The mean Cohen's kappa value obtained was 0.91, (range: 0.79-1.00), indicating excellent levels of coder agreement (8).

ADDITIONAL RESULTS

Detailed description of Fiona-Sutherland showing event

This event was observed by CW on the 18th May 2019 at 15:27 while she followed a party of chimpanzees at Ngogo. Fiona, a 22-year old adult female, was seated on the ground next to her mother, Sutherland (~56 years old). Fiona had been grooming Sutherland in the 30s before video recording began, and appeared to be manipulating an object in her mouth/on her lips; this was likely an ectoparasite she found while grooming Sutherland. Fiona proceeded to pick up a small leaf and apparently placed the object on the leaf with her lips. Leaf grooming as a way of inspecting ecto-parasites has been documented at other sites (*Tai*, (9); *Mahale,* (10); *Budongo*, (11)). Fiona proceeded to groom this leaf for 8 seconds, manually manipulating and folding it. She then held it between her lips while reaching to a nearby sapling to pluck another, bigger, leaf. Once she had the new leaf, she took the old leaf from her mouth, looked at it, and then discarded it. She then appeared to place the object on the new leaf using her lips. She folded the leaf once, then held it out towards Sutherland, with her arm bent, and seemed to glance at Sutherland's face. Until that point Sutherland had not looked at Fiona or the leaves, but she now looked down at the leaf. Fiona adjusted her arm twice more, ending with her arm being fully extended and the leaf directly in front of Sutherland, making it virtually impossible for Sutherland to miss seeing it. Sutherland kept her gaze on the leaf and followed its movement with her head; she was clearly aware of it and attending to it by the time it reached its final position. Throughout this 'showing' movement the camera angle made it impossible to accurately track Fiona's gaze, but her head direction indicated she was looking at both the leaf and Sutherland's face. Once Sutherland had moved her head in line with the leaf, Fiona retracted her arm and resumed leaf-grooming (she groomed the second leaf for a total of 29s and then a third leaf for another 18s). Sutherland continued to sit and look elsewhere, then lay down to rest 33s after the end of the 'showing'. Fiona resumed grooming Sutherland 9s after this, once she had discarded her final leaf.

Legend for Video S1

Video S1. Video presenting the showing gesture produced by Fiona to Sutherland at normal speed, then in slow motion with subtitles to label the important behaviours being shown.

Leaf-grooming events

A leaf-grooming event started when a new leaf was picked up or plucked, and ended when this leaf was discarded.

Eighty-four full leaf-grooming events (63 from Kanyawara, 21 from Ngogo; N=37 leaf-groomers, each contributing 1-12 events, were identified in which at least one individual was within 1m and in sight. For 30 of these 84 events there was only one individual within 1m, and the remainder had multiple individuals within 1m (overall range: 1-5). At least one subadult or adult individual (>10 years) was

within 1m of the leaf-groomer in 66 events. At least one other individual was within 1m and could observe the leaf-grooming without having to move their torso in 78 events.

The youngest recorded leaf-groomer was 5 years old. We considered only individuals >1 year old as potential partners for the leaf-groomers, that is, as potentially attending visually to the leaf-grooming event.

The average duration of leaf-grooming events was 30.2s (SD=26.4), with a range of 5-137s.

Other individuals observing the leaf-grooming event (peering/watching)

We could ascertain whether at least one other individual watched or peered in 74 of the 78 events in which at least one other individual was within 1m and could have attended visually without moving their torsos.

From the leaf-groomer's perspective, at least one other individual within 1m watched or peered at their activity, creating simultaneous visual attention to the leaf, in 60/74 events (81.1%, N=32 leaf-groomers). From the audience perspective, across the 74 events there were 138 individuals within 1m who were able to potentially see the leaf-grooming without moving their torso. The filming angle made it impossible to code whether 4/138 individuals watched or peered, so we did not consider these individuals further. When an individual watched *and* peered we coded this as peering. Forty-five of the remaining 134 potential observers (33.6%) peered (defined as face <30cm from the leaf), 47 (35.1%) watched (defined as attending visually with face >30cm from the leaf), and 42 (31.3%) did not attend visually.

Thus, when spatial constellations mean it is easy for potential observers to notice and visually attend to leaf-grooming, simultaneous visual attention on the leaf-grooming occurred in the majority of events, suggesting that leaf-grooming draws the interest and attention of others. This is in line with similar observations of other individuals attending to leaf-grooming, that have been made at Mahale (12). Remarkably, in our data set, about a third of potential observers approached to peer at the leaf closely. When they are in such close physical proximity, both the leaf-groomer and the individual peering are almost certainly aware that they are attending to the same thing, creating an ideal situation for the sharing of attention (13).

Leaf-groomer behaviour immediately before and after leaf-grooming

The behaviour of the leaf-groomer both immediately before (5s) and immediately after (5s) could be recorded for 58 events (N=30 leaf-groomers). There were 36 events where the leaf-groomer was engaging in a non-social activity immediately before the leaf-grooming event (i.e. resting, leaf-grooming a different leaf, self-grooming, feeding); for 32 of these the leaf-groomer continued engaging in a non-social activity following the leaf-grooming event, in the remaining four cases they were subsequently groomed by another individual. There were 22 events where the leaf-groomer was engaged in a social activity (grooming with another individual) immediately before the leaf grooming event. There were 18 events where the leaf-groomer was grooming another individual prior to the leaf-grooming event; following the event they continued grooming the other individual in six cases, were groomed by the other individual in three cases, self-groomed in three cases, leaf-groomed again in three cases, and played with another individual in one case. There were three events where the leaf-groomer was being groomed by another individual prior to the event;

following the event they continued being groomed in one case, groomed the other in one case, and self-groomed in one case. There was one case where the leaf-groomer was engaging in mutual grooming before the event, and then rested following the event. There were no cases where the leaf-groomer was playing prior to the event.

REFERENCES

1. R.W. Wrangham,C. A. Chapman,A. P. Clark-Arcadi,G. Isabirye-Basuta, “Social ecology of Kanyawara chimpanzees: implications for understanding the costs of great ape groups” in Great Ape Societies, W. C. Mcgrew, L. Marchant, T. Nischida, Eds. (Cambridge University Press, 1996), pp. 45–57.
2. C. A. Chapman,J. E Lambert, Habitat alteration and the conservation of African primates: Case study of Kibale National Park, Uganda. *Am. J. Primatol.,* **50**, 169–185 (2000).
3. T. T. Struhsaker, *Ecology of an African rain forest: logging in Kibale and the conflict between conservation and exploitation* (University Press of Florida, 1997).
4. R. W. Wrangham,A. P. Clark,G. Isabirye-Basuta, Female social relationships and social organization of Kibale Forest chimpanzees. *Topics in Primatatology, 1(Human Origins),* 81–98 (1992).
5. M. E. Thompson, M. N. Muller, Z. P. Machanda, E. Otali, R. W. Wrangham, The Kibale Chimpanzee Project: Over thirty years of research, conservation, and change. *Biol. Conserv.* **252**, 108857 (2020).
6. J. C. Mitani,D. P. Watts, Demographic influences on the hunting behavior of chimpanzees. *Am. J. Phys. Anthropol.* **109***,* 439–454 (1999).
7. A. A. Sandel,D. P. Watts, Lethal Coalitionary Aggression Associated with a Community Fission in Chimpanzees (*Pan troglodytes*) at Ngogo, Kibale National Park, Uganda. *Int. J. Primatol.*, **42**, 26–48 (2021).
8. J. L. Fleiss, Balanced Incomplete Block Designs for Inter-Rater Reliability Studies. *Appl. Psychol. Meas.* **5**, 105–112 (1981).
9. C. Boesch, Innovation in wild chimpanzees (*Pan troglodytes*). *Int. J. Primatol*. **16**, 1-16 (1995).
10. K. Zamma, Leaf-grooming by a wild chimpanzee in Mahale. *Primates* **43**, 87-90 (2002).
11. C. Assersohn,A. Whiten,Z. T. Kiwede,J. TinkaJ. Karamagi, Use of leaves to inspect ectoparasites in wild chimpanzees: a third cultural variant?. *Primates* **45**, 255-258 (2004).
12. K. Zamma,M. Nakamura, “Grooming: Its hygienic and social aspects” in Mahale Chimpanzees: 50 Years of Research, M. Nakamura, K. Hosaka, N. Itoh, K. Zamma, Eds. (Cambridge University Press, 2015), pp. 472-484.
13. B. Siposova,M. Carpenter, A new look at joint attention and common knowledge. *Cognition* **189**, 260-274 (2019).
